# REST and CoREST Modulate Neuronal Subtype Specification, Maturation and Maintenance

**DOI:** 10.1371/journal.pone.0007936

**Published:** 2009-12-07

**Authors:** Joseph J. Abrajano, Irfan A. Qureshi, Solen Gokhan, Deyou Zheng, Aviv Bergman, Mark F. Mehler

**Affiliations:** 1 Institute for Brain Disorders and Neural Regeneration, Albert Einstein College of Medicine, Bronx, New York, United States of America; 2 Department of Neurology, Albert Einstein College of Medicine, Bronx, New York, United States of America; 3 Department of Neuroscience, Albert Einstein College of Medicine, Bronx, New York, United States of America; 4 Department of Psychiatry and Behavioral Sciences, Albert Einstein College of Medicine, Bronx, New York, United States of America; 5 Department of Pathology, Albert Einstein College of Medicine, Bronx, New York, United States of America; 6 Department of Genetics, Albert Einstein College of Medicine, Bronx, New York, United States of America; 7 Department of Systems and Computational Biology, Albert Einstein College of Medicine, Bronx, New York, United States of America; 8 Einstein Cancer Center, Albert Einstein College of Medicine, Bronx, New York, United States of America; 9 Rose F. Kennedy Center for Research on Intellectual and Developmental Disabilities, Albert Einstein College of Medicine, Bronx, New York, United States of America; Tokyo Medical and Dental University, Japan

## Abstract

**Background:**

The repressor element-1 silencing transcription factor/neuron-restrictive silencer factor (REST/NRSF) is a master regulator of neuronal gene expression. REST functions as a modular scaffold for dynamic recruitment of epigenetic regulatory factors including its primary cofactor, the corepressor for element-1-silencing transcription factor (CoREST), to genomic loci that contain the repressor element-1 (RE1) binding motif. While REST was initially believed to silence RE1 containing neuronal genes in neural stem cells (NSCs) and non-neuronal cells, emerging evidence shows an increasingly complex cell type- and developmental stage-specific repertoire of REST target genes and functions that include regulation of neuronal lineage maturation and plasticity.

**Methodology/Principal Findings:**

In this study, we utilized chromatin immunoprecipitation on chip (ChIP-chip) analysis to examine REST and CoREST functions during NSC-mediated specification of cholinergic neurons (CHOLNs), GABAergic neurons (GABANs), glutamatergic neurons (GLUTNs), and medium spiny projection neurons (MSNs). We identified largely distinct but overlapping profiles of REST and CoREST target genes during neuronal subtype specification including a disproportionately high percentage that are exclusive to each neuronal subtype.

**Conclusions/Significance:**

Our findings demonstrate that the differential deployment of REST and CoREST is an important regulatory mechanism that mediates neuronal subtype specification by modulating specific gene networks responsible for inducing and maintaining neuronal subtype identity. Our observations also implicate a broad array of factors in the generation of neuronal diversity including but not limited to those that mediate homeostasis, cell cycle dynamics, cell viability, stress responses and epigenetic regulation.

## Introduction

The repressor element-1 silencing transcription factor/neuron-restrictive silencer factor (REST/NRSF) is a transcriptional regulator with genome-wide effects important for orchestrating neuronal development [1]. REST binds to repressor element-1 (RE1) consensus motifs [2] which are primarily located in promoter regions of genes responsible for fundamental mature neuronal traits including: ion channels, adhesion molecules, synaptic vesicle proteins, growth factors and hormones, axonal guidance and vesicle trafficking, and neurotransmitter receptors [3], [4], [5]. While REST was initially believed to repress transcription of these neuronal genes in neural stem cells (NSCs) and in non-neuronal cells, recent evidence suggests a much broader role for REST, with context-specific and sometimes seemingly paradoxical functions in embryonic stem cells (ESCs), NSCs, mature neurons, and other cell types [3], [5], [6].

The differential roles played by REST depend on its ability to recruit a series of epigenetic and regulatory cofactors to its N- and C-terminal domains. These highly plastic macromolecular complexes often include another important transcriptional regulator, the corepressor for element-1-silencing transcription factor (CoREST), the primary cofactor of REST [7]. Like REST, CoREST recruits additional epigenetic factors similarly associated with gene activation and repression, including methyl CpG binding proteins (e.g., MeCP2), histone deacetylases (e.g., HDAC1/2), histone modifying enzymes (e.g., LSD1, EHMT2/G9a, and SUV39H1), and components of SWI-SNF chromatin remodeling complexes (e.g., BAF57, BRG1, and BAF170) [8], [9], [10], [11].

Various studies have begun to characterize the specific roles played by REST and CoREST complexes during NSC-mediated neuronal lineage specification and maturation. For example, distinct REST and CoREST complexes were found to regulate target gene expression in post-mitotic neurons [12], [13]. In these studies, for a subset of targets designated as class I neuronal genes, a maximum level of expression was observed with REST complex dissociation from the gene promoter. For class II genes, a submaximal level of expression was found when the REST complex dissociated from the gene promoter due to the presence of a separate CoREST complex at a different promoter site [12]. In addition, other studies have also started to describe the specific roles played by cofactors in these complexes. During adult hippocampal neurogenesis, REST is converted from a transcriptional repressor into an activator by a small modulatory double stranded RNA (dsRNA) [14], [15]. Moreover, a truncated isoform of REST, REST4, exerts a dominant-negative effect on REST and possibly derepresses or activates expression of RE-1 containing genes in neurons [15]. Additional studies have identified cell type-specific profiles of REST target genes and suggested that many REST target genes and functions have yet to be discovered [16], [17], [18], [19].

In this study, we examined the roles of REST and CoREST during NSC-mediated neuronal subtype specification and maintenance. While these processes are critical for establishing functional diversity, homeostasis, and neural network connectivity and plasticity within the nervous system [20], the regulatory circuitry responsible for governing the elaboration of different neuronal subtypes remains poorly understood. We found specific REST and CoREST target gene profiles in neuronal subtypes, including cholinergic neurons (CHOLNs), GABAergic neurons (GABANs), glutamatergic neurons (GLUTNs), and medium spiny projection neurons (MSNs)], including those not previously associated with the establishment of neuronal subtype identity, such as particular epigenetic factors, cell cycle regulators and homeostatic modulators. These observations suggest that the complex gene networks underlying neuronal subtype specification and maintenance are selectively modulated by the differential deployment of REST and CoREST.

## Results

We examined the molecular mechanisms that underlie neuronal subtype specification by characterizing REST and CoREST protein expression and target gene profiles using ChIP-chip analysis in a developmental paradigm associated with the elaboration of mature neuronal subtypes. We identified REST and CoREST target genes that are unique to a specific neuronal subtype and also those found in multiple subtypes. In addition, we interrogated the potential roles of REST and CoREST in regulating gene expression during neuronal subtype specification by examining correlations between profiles of REST and CoREST promoter occupancy and corresponding target gene expression patterns during critical developmental transitions.

### Identification of Dorsal and Ventral Forebrain Developmental and Mature Cellular Species by the Expression of Selective Neural Lineage Markers and Associated Transcription Factor Codes

Both dorsal and ventral NSCs are identified by their expression of the neuroepithelial marker, nestin in the absence of expression of other lineage markers associated with intermediate neural progenitors and neuronal or glial species (Figure S1). Radial glial cells derived from dorsal forebrain NSCs continued to express nestin while acquiring RC2 [21] (Figure S1). N/OPs are defined by their expression of two basic helix-loop-helix transcription factors, Olig2 and Mash1 [22] in addition to presence of the neuropeithelial marker, nestin (Figure S1). GLUTN derived from RG cells [23], [24] acquired expression of the early neuronal linage marker, β tubulin III and exhibited complete overlap with the excitatory neurotransmitter, glutamate, while ventral forebrain derived neuronal species were identified by their co-expression of β tubulin III in concert with acquisition of distinct neurotransmitters (GABA for GABAN) or substrates and enzymes required for the processing and synthesis of neuronal subtype specific neurotransmitters (DARPP32, MSN and ChAT, CHOLN, respectively) [25] (Figure S1).

### Determination of REST and CoREST Expression and Subcellular Localization

We characterized REST and CoREST protein expression and localization during the process of neuronal subtype specification using immunofluorescence microscopy and Western blot analysis (Figures S2, S3, S4). We found that both proteins displayed more nuclear expression in NSCs and the intermediate progenitors, RGs and N/OPs, while being differentially distributed to the nucleus and cytoplasm of all neuronal subtypes examined (Figures S2, S3, S4). These observations suggest that both REST and CoREST are present in all neuronal subtypes and therefore have the potential to regulate gene expression during neuronal subtype specification.

### Genome-Wide Promoter Occupancy Profiles for REST and CoREST

**Figure 1:**
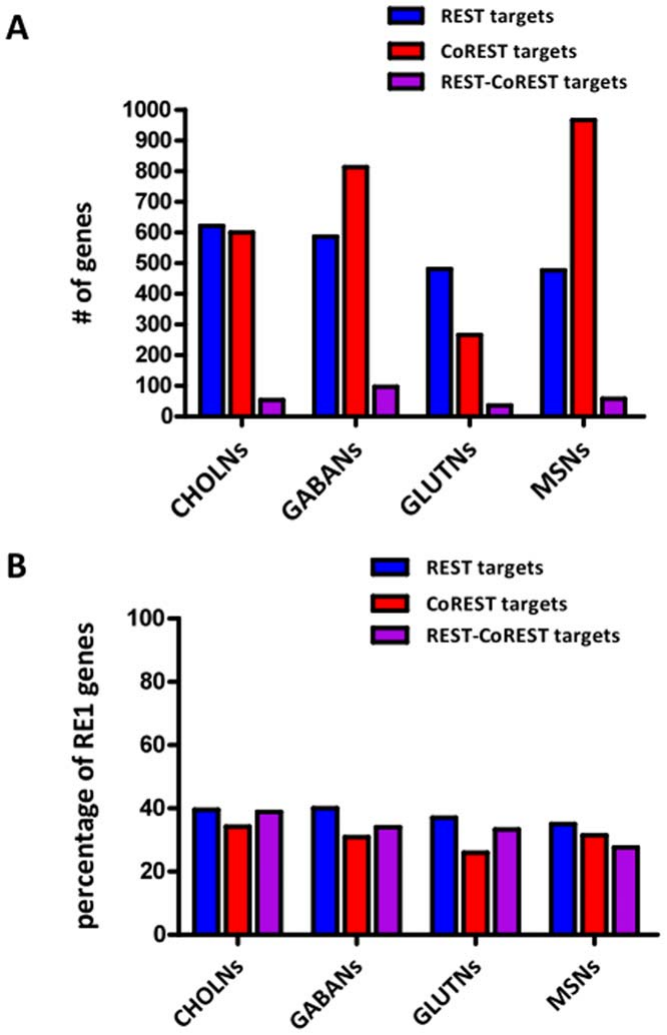
Profiles of REST and CoREST target genes in neuronal subtypes. (A) The number of exclusive REST, exclusive CoREST, and REST and CoREST (REST-CoREST) target genes uniquely present in individual neuronal subtypes as identified through chromatin immunoprecipitation on promoter chip (ChIP-chip) experiments. We identified 2,123 total REST target genes and 2,384 total CoREST target genes. (B) The percentages of REST, CoREST, or REST-CoREST target genes in individual neuronal subtypes that contain previously characterized repressor element-1 (RE1) motifs [16], [18].

While REST and CoREST expression and localization remain relatively constant, there is significant variation in the number of gene promoter sites occupied across the different neuronal subtypes (Figure 1A). We found a total of 2,123 REST and 2,384 CoREST target genes across all neuronal cell types examined. Amongst these genes, we found that 1,453 are “exclusive” targets of REST, 1,714 are exclusive targets of CoREST, and 670 are targets of both REST and CoREST (REST-CoREST). We designated these genes as “unique” targets if they were bound only within one of the neuronal subtypes examined. For CHOLNs, GABANs, GLUTNs, and MSNs, we identified 622, 587, 481, and 477 unique and exclusive REST target genes; and we found 600, 814, 266, and 967 unique and exclusive CoREST target genes. For CHOLNs, GABANs, GLUTNs, and MSNs, we further identified 54, 97, 36, and 58 unique REST-CoREST target genes. These observations show that profiles of REST, CoREST, and REST-CoREST target genes are largely unique and vary significantly in number across different neuronal subtypes, suggesting that REST and CoREST may perform diverse and differential functions during neuronal subtype specification.

We also compared the REST and CoREST target genes we identified across the four neuronal subtypes with a previously identified set of canonical and non-canonical RE1 sequence containing genes (Figure 1B) [16], [18]. Interestingly, for the subgroups of genes that are REST, CoREST, and REST-CoREST targets, the percentages of RE1 containing genes range only from 30 to 40%. Our results also show that, in these neuronal subtypes, REST and CoREST principally target genes that have not previously been found to contain RE1 motifs. These observations suggest that REST and CoREST genomic interactions are highly complex and provide further support for the existence of a neuronal lineage-specific regulatory hierarchy for REST and CoREST that employs differential DNA binding motifs to encode functional information [19].

**Figure 2:**
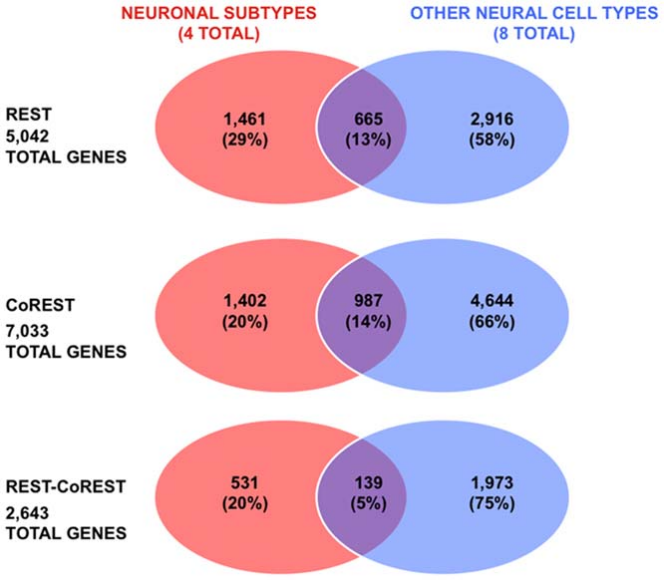
Comparative analysis of REST, CoREST, and REST-CoREST target gene profiles in neuronal subtypes and other neural cell types. The distinct and overlapping profiles of REST, CoREST and REST-CoREST target genes present in neuronal subtypes and in all other neural cell types. We compared profiles of REST and CoREST target genes in neuronal subtypes [cholinergic neurons (CHOLNs), medium spiny projection neurons (MSNs), GABAergic neurons (GABANs), glutamatergic neurons (GLUTNs)] with other neural cell types [neural stem cells, neuronal/oligodendrocyte (OL) precursors, radial glia, astrocytes, OL precursors, OL progenitors, post-mitotic OLs, and myelinating OLs]. Note the relatively small number of REST and CoREST target genes found in common between these neural species.

To begin characterizing the roles of REST and CoREST in modulating neuronal subtype specific gene networks, we compared the profiles of REST and CoREST target genes from all neuronal subtypes to those from a corresponding study we performed in a subset of immature neural and macroglial cell types (Figure 2). Unexpectedly, we observed that these composite profiles are quite distinct from each other. We found that only 13% of REST target genes in neuronal subtypes are also REST targets in the other cell types, 14% of CoREST target genes in neuronal subtypes are also CoREST targets in the other cell types, and 5% REST-CoREST target genes in neuronal subtypes are also REST-CoREST targets in the other cell types.

### Pathway Analysis of REST and CoREST Target Genes

To compare the functional roles of REST and CoREST, we analyzed profiles of REST and CoREST target genes in neuronal subtypes using Ingenuity Pathway Analysis and found a myriad of enriched pathways that may play roles in neuronal subtype elaboration (Table 1). For exclusive REST target genes, these pathways include hypoxia signaling. In contrast, for exclusive CoREST target genes, these pathways include CD28 signaling, chemokine signaling, glucocorticoid receptor signaling, and Huntington's disease signaling. Also, for exclusive REST target genes and exclusive CoREST target genes, these pathways include the protein ubiquitination and estrogen receptor signaling. Finally, for REST-CoREST target genes, these pathways include tight junction signaling, induction of apoptosis, protein ubiquitination, PTEN signaling, and neuregulin signaling. These results indicate that REST, CoREST, and REST-CoREST coordinately regulate a diverse array of neuronal homeostasis pathways involved in stress responses, cell cycle progression, and trophic factor signaling and suggest that REST and CoREST perform similar functional roles through different subsets of target genes.

**Table 1:** Comparative analysis of pathways enriched in composite profiles of REST, CoREST and REST-CoREST target genes.

Ingenuity Canonical Pathway	REST	CoREST	REST-CoREST
Hypoxia Signaling in the Cardiovascular System	3.36		
Estrogen Receptor Signaling	2.49	3.07	
Protein Ubiquitination Pathway		2.44	2.17
CCR3 Signaling in Eosinophils		3.36	
IL-8 Signaling		3.23	
CXCR4 Signaling		3.16	
fMLP Signaling in Neutrophils		2.88	
LPS-stimulated MAPK Signaling		2.63	
Macropinocytosis		2.45	
CD28 Signaling in T Helper Cells		2.43	
Role of NFAT in Regulation of the Immune Response		2.41	
Chemokine Signaling		2.41	
T Cell Receptor Signaling		2.39	
Ephrin Receptor Signaling		2.35	
Cardiac Hypertrophy Signaling		2.27	
Thrombin Signaling		2.25	
Glucocorticoid Receptor Signaling		2.23	
Renin-Angiotensin Signaling		2.04	
Huntington's Disease Signaling		2.03	
Tight Junction Signaling			2.68
Induction of Apoptosis by HIV1			2.33
PTEN Signaling			2.17
Neuregulin Signaling			2.15

The values in each row represent the degree of pathway enrichment (–LogP) for each set of target genes. Only pathways exhibiting high statistical significance (–LogP>2) are included. Target genes were analyzed using Ingenuity Pathways Analysis (Ingenuity® Systems, www.ingenuity.com).

### Pathway Analysis of Target Genes in Individual Neuronal Subtypes

To further examine the distinct and overlapping roles for REST and CoREST within neuronal subtypes, we examined REST and CoREST target genes in four mature neuronal subtypes (CHOLNs, GABANs, GLUTNs, and MSNs) (Table 2). We found that REST target genes are involved in a diverse set of biological functions. Specifically, in CHOLNs, these pathways include IL-17 signaling and C21-steroid hormone metabolism. In GABANs, these pathways include p38 MAPK signaling and arachidonic acid metabolism. In contrast, CoREST target genes are involved in a more delimited set of biological functions. For example, in MSNs, these pathways include nitrogen metabolism and aminosugar metabolism. In CHOLNs these pathways include riboflavin metabolism and estrogen receptor signaling. Finally, in GABANs, these pathways include complement system and cell cycle: G2/M DNA damage checkpoint regulation.

**Table 2:** Comparative analysis of pathways enriched in REST and CoREST target genes for individual neuronal subtypes.

Ingenuity Canonical Pathway	Analysis	Enrichment (-LogP)
C21-Steroid Hormone Metabolism	REST MSN	1.51
Fc Receptor-mediated Phagocytosis in Macrophages and Monocytes	REST GLUTN	2.60
Neuregulin Signaling	REST GLUTN	2.48
Natural Killer Cell Signaling	REST GLUTN	2.20
CTLA4 Signaling in Cytotoxic T Lymphocytes	REST GLUTN	2.15
CXCR4 Signaling	REST GLUTN	1.66
Fc Epsilon RI Signaling	REST GLUTN	1.52
Estrogen Receptor Signaling	REST GLUTN	1.34
CCR5 Signaling in Macrophages	REST GLUTN	1.33
p38 MAPK Signaling	REST GABAN	1.47
Arachidonic Acid Metabolism	REST GABAN	1.47
Hypoxia Signaling in the Cardiovascular System	REST GABAN	1.37
Nitrogen Metabolism	CoREST MSN	1.97
Aminosugars Metabolism	CoREST MSN	1.85
Lymphotoxin β-Receptor Signaling	CoREST GLUTN	1.54
Complement System	CoREST GABAN	1.50
Cell Cycle: G2/M DNA Damage Checkpoint Regulation	CoREST GABAN	1.34
Riboflavin Metabolism	CoREST CHOLN	1.68
Estrogen Receptor Signaling	CoREST CHOLN	1.62
PI3K/AKT Signaling	REST CHOLN	2.27
Biosynthesis of Steroids	REST CHOLN	2.10
IL-17 Signaling	REST CHOLN	1.73
Hypoxia Signaling in the Cardiovascular System	REST CHOLN	1.39
Pyrimidine Metabolism	REST CHOLN	1.37
C21-Steroid Hormone Metabolism	REST CHOLN	1.31

The value in each row represents the degree of pathway enrichment (–LogP) for each set of target genes. Only pathways exhibiting statistical significance (–LogP>1.3) are included. Target genes were analyzed using Ingenuity Pathways Analysis (Ingenuity® Systems, www.ingenuity.com).

### Roles of REST and CoREST in Neuronal Subtype Identity, Maintenance, and Function

#### Cholinergic neurons

Cholinergic neurons (CHOLNs) are basal forebrain excitatory neurons that provide the major cholinergic input to the neocortex [26]. Our results suggest that a subset of REST and CoREST target genes (Table S1) modulate aspects of CHOLN specification and maintenance, including those involved in gradient morphogen signaling (e.g., *Bmp2*), cellular processes (e.g., *Smad2*), transcriptional regulation (e.g., *Lhx8*), and cell-cell signaling (e.g., *Cnr1*). Specifically, bone morphogenetic protein (BMP) signaling plays an important role during various stages of neural development including the differentiation of cortical progenitors into distinct neuronal subtypes [27]. While Bmp2 promotes CHOLN specific gene expression in the basal forebrain [28], Smad2 is essential for ventral forebrain patterning and has been shown to regulate excitability of CHOLNs [29], [30], [31]. In addition, REST targets the LIM homeobox transcription factor Lhx8, which is selectively expressed in the embryonic medial ganglionic eminence (MGE), where it is required for CHOLN maintenance in the developing basal forebrain and may also play a role in establishing CHOLNs rather than GABANs [32], [33], [34]. Finally, Cnr1 is important for CHOLN specification and long-range axonal patterning [35]. Our cumulative evidence demonstrates that REST and CoREST modulate an array of target genes, including those with roles in CHOLN morphology and maintenance as well as other genes that actively suppress the elaboration of alternate neuronal subtypes.

#### GABAergic neurons

We identified target genes with diverse roles in forebrain specification and development, including genes involved in transcriptional regulation (e.g., *Dlx2*, *Mib1*, and *Bhlhb5*), neuronal subtype specification (e.g., *Neo1* and *Gabrg1*), and neuroblast proliferation and differentiation (e.g., *Rarβ* and *Elavl2*). Dlx2 is a homeobox factor that promotes the specification of GABANs [36]. We also identified Mib1, an ubiquitin ligase necessary for efficient Notch signaling that specifies GABANs through interactions with the Dlx family of homeobox transcription factors [37]. Further, we identified other factors with specific roles in GABAN differentiation, including Neo1, a netrin receptor that plays a role in the tangential migration of cortical GABANs [38], and Bhlhb5, a basic helix-loop-helix (bHLH) factor that is induced during the intermediate phase of the Ngn2-regulated cortical differentiation cascade that determines the decision to differentiate between GLUTNs and GABANs [39]. Finally, besides specific roles in GABANs, we also found that REST and CoREST mediate neuronal homeostasis programs. Specifically, sequential Rarβ and Rarα signaling can lead to the proliferation and differentiation of forebrain progenitors in concert with Shh and FGF signaling pathways [40]. Elavl2 is a neuronal RNA binding protein that is a target of Ngn2 and is important for controlling the switch from proliferation to neuronal differentiation through the post-transcriptional regulation of p21^Cip1/Waf1^ [41], [42]. Our results suggest that REST and CoREST mediate factors directly involved in diverse aspects of GABAN subtype specification and terminal differentiation as well as other factors with roles in neuronal homeostasis programs. These findings are consistent with evolving evidence that homeostatic factors, such as cell cycle regulators, are critical in post-mitotic neuronal functions [43].

#### Glutamatergic neurons

We found target genes critical for GLUTN specification and forebrain patterning, including those involved in neuronal morphogenesis and plasticity (e.g., *Ctnnd2*), transcriptional regulation (e.g., *Tlx1*), and cytokine signaling (e.g., *Bmp2* and *Lif*). We identified Ctnnd2, a factor essential for the trafficking and proper localization of glutamate receptors (GluR2) in dendritic spines [44]. Also, Tlx1 is a homeobox transcription factor that promotes the specification of GLUTNs and inhibits the acquisition of GABANs by inhibiting the effects of Lbx1, a GABAN differentiation factor [45]. We also identified genes encoding factors directly involved in the specification of GLUTNs from RG [46] including BMP2, which promotes lineage restriction of RG into GLUTNs in concert with LIF [21], an interleukin-6 (IL6) family cytokine.

We further identified target genes with roles in forebrain development, including those involved in neuroblast proliferation (e.g., *Pten1*), lineage commitment (e.g., *Gjc1*), maturation (e.g., *Dcp1a* and *Smad2*), and synaptogenesis (e.g., *Akt1*). While Pten promotes endogenous NSC expansion and self-renewal [47], Gjc is selectively expressed in committed neuronal progenitor cells [48], [49], [50]. We also identified mediators of TGF-β signaling, including BMP-related pathways, such as Dcp1a and Smad2, both of which are involved in neural patterning, specification and maturation. Finally, Akt1 is involved in aspects of prefrontal cortex architecture, including neuronal morphology, synaptic function, and axonal myelination [51]. These cumulative results suggest that REST and CoREST regulate factors with specific roles in defining GLUTN specification and maintenance. Our results also suggest that neuronal maturation is intrinsically linked to neuronal subtype specification and that these fundamental developmental processes are, in large part, coordinately regulated by REST and CoREST.

#### Medium spiny projection neurons

We identified target genes involved in promoting MSN morphogenesis (e.g., *Cobl*), maturation and subtype specific functions (e.g., *Mash1*, *Nts* and *Grm5*). Mash1 is a proneural bHLH member that promotes neural precursor cell cycle exit and differentiation [52]. Nts is a neuropeptide expressed in MSNs that is important for striatal activity [53], and Grm5 is a metabotropic glutamate receptor densely expressed in the spines of MSNs [54]. Intriguingly, cortical glutamate and substantia nigra dopamine (DA) afferents converge onto striatal MSN dendritic spines where they modulate motor and cognitive functions.

We further identified target genes that have roles in neuronal morphogenesis (e.g., *Evl* and *Nefm*) and migration (e.g., *Plxnb1*). Evl, an actin regulatory protein, is highly expressed in the developing cortical plate and regulates cortical neuronal positioning [55], [56], while Nefm is a neurofilament subunit that modulates cytoskeletal stability and determines cortical neuronal functions [57]. Also, Plxnb1 is a semaphorin 4D receptor widely expressed in the developing neopallial cortex that regulates neuronal migration [58]. Finally, we found gene targets involved in both pan-neuronal (e.g., *Purb* and *Tbca*) and neural (e.g., *Egfr*) differentiation. Purb, a nucleic acid binding protein similar to Pura, controls DNA replication and transcription, mediates dendritic translocation of ncRNAs and mRNAs, and assists in establishing the postsynaptic compartment in developing neurons [59]. Tbca promotes proper folding of a pan-neuronal protein, β-tubulin [60]. Also, EGFR-mediated signaling pathways appear to have diverse roles in neuronal subtype specification [61]. Specifically, asymmetric distribution of EGFR contributes to forebrain development by creating progenitors with different apoptotic, proliferative, migratory, and differentiation responses to ligand [61], [62], [63], [64]. These cumulative findings suggest that REST and CoREST regulate factors associated with MSN-specific morphology and differentiation. They also indicate that REST and CoREST differentially regulate the deployment of gene networks preferentially involved in mediating broader developmental and homeostatic programs during neuronal subtype specification.

### Roles of REST and CoREST in Developmental Processes

#### Epigenetic factors

**Figure 3:**
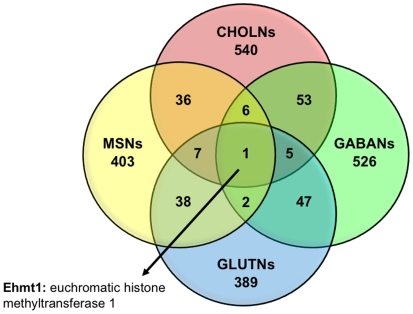
Comparative profiles of REST target genes present in neuronal subtypes. We examined the distinct and overlapping profiles of REST target genes present in individual neuronal subtypes.

**Figure 4:**
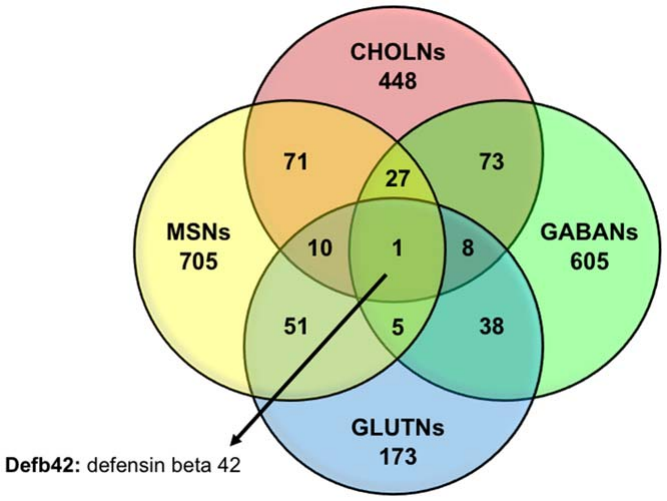
Comparative profiles of CoREST target genes present in neuronal subtypes. We examined the distinct and overlapping profiles of CoREST target genes present in individual neuronal subtypes.

We examined both common and unique gene targets for REST and CoREST within mature neuronal subtypes (Figures 3 and 4). Interestingly, the common REST and CoREST targets we identified in all four mature neuronal subtypes are directly or indirectly involved or related to EHMT2/G9a, a H3K9 histone methyltransferase (HMT) primarily associated with heterochromatic regions and recruited directly to the REST complex by CoREST [65], [66]. Specifically, REST targets EHMT1, a G9a related HMT that mediates gene silencing through mono- and dimethylation of H3K9 [66], [67], [68]. This HMT may, in fact, be an integrative factor critical for maintaining epigenetic states that link histone modifications with DNA methylation states previously shown to be mediated by components of the REST repressor complex [10], [69]. Furthermore, CoREST targets the gene locus for Defb42, a G9a-regulated factor that is a member of the defensin family, which is implicated in mediating innate and acquired immunity and may be important for immune surveillance in the nervous system [67], [68].

We also identified a diverse array of REST and CoREST targets involved in epigenetic processes (Table S2), including genes that mediate DNA methylation states (e.g., *Mbd6*), histone modifications (e.g., *Smcx*, *Jarid1d*, *Jmjd1a*, and *Lsd1*), and the SWI/SNF family of chromatin remodeling enzymes (e.g., *Smarca5*, *Smarcc1*, *Smarcc2*, and *Smarce1*). A number of these factors have previously been associated with the REST complex and its functions, which range from maintenance of higher-order chromosomal organization and stability to DNA methylation, nucleosome dynamics, and chromatin remodeling [7], [10]. Specifically, we identified Jarid1c, a H3K4me3 demethylase recruited by the REST complex that is essential for neuronal survival and dendritic development [70]. Jarid1c mutations are associated with both X-linked MR and autism spectrum disorder [71], [72]. Moreover, we identified components of dynamic combinatorial SWI/SNF nucleosome remodeling complexes, including subunits of the Brg1/Brm Associated Factors (BAF) complex (e.g., *Smarce1* and *Smarcc1*) and imitation SWI (ISWI) members (e.g., *Smarca5*, *Baz1b*, and *Smarca1*). Interestingly, Smarce1 and Smarcc1 can heterodimerize and associate with SWI/SNF complexes in combination with Smarca4, Smarca2, and Smarcc2. Also, CoREST recruits Smarce1, which has neuron-specific isoforms that are differentially expressed during neurogenesis and are believed to play specific roles in establishing neuronal subtype identity [73]. Similarly, ISWI members play important roles in brain development through effects on transcriptional regulation, heterochromatin replication, chromatin assembly, and chromatin higher-order structure [74]. Furthermore, Snf2h forms a complex with Wstf and is implicated in neural progenitor cell proliferation, while Snf2l has gene-specific effects during neuronal terminal differentiation [75]. These cumulative findings suggest that REST and CoREST mediate deployment of various classes of epigenetic factors in neuronal subtypes, thereby, suggesting important roles for these factors in regulating multi-layered epigenetic cascades during neuronal subtype specification.

#### Cell cycle, ubiquitination, and apoptosis

A number of REST and CoREST target genes encode factors involved in cell cycle regulation (Table S3), including Mad2, an essential component of proper spindle checkpoint assembly that is regulated by ubiquitin ligase SCF(beta-TrCP)-dependent degradation of REST during the G2 phase of the cell cycle [76]. We also identified key factors involved in the ubiquitin-proteosome pathway (Table S4), including Cul1, a scaffolding protein for SCF ubiquitin ligases implicated in the regulation of cell cycle progression, synaptic morphology and function, and neuronal apoptosis [77], [78]. Furthermore, we uncovered a subset of other apoptotic factors (Table S5), including Casp1, a modulator of neuronal apoptosis which acts as part of a viability rheostat [79], [80]. In concert with recent studies, our results indicate that REST and CoREST coordinate neuronal subtype specific gene expression programs by modulating factors involved in cell cycle regulation [43] and protein turnover [81] as well as those that orchestrate the sequential developmental elaboration of mature, post-mitotic neurons.

#### Neuronal identity factors

We found that REST and CoREST modulate genes encoding neuronal identity factors that regulate various aspects of forebrain development (Table S6). For example, Jag1 is a Notch ligand that promotes seminal neural fate decisions [82], suggesting REST and CoREST modulate developmental signaling pathways important for neuronal subtype specification. Jag1 also promotes microtubule stability and axon integrity [83]. In addition, Neo1 is a netrin receptor involved in axon pathfinding and regulation of neuronal differentiation [84]. Our findings thereby suggest a role for REST and CoREST in modulating axonal processes and plasticity in neuronal subtypes, which is supported by previous evidence showing that misexpression of REST causes axon guidance errors [85].

We identified factors with roles in transcriptional regulation of forebrain patterning. For example, NeuroD4/Math3 is a dorsal bHLH transcription factor that cooperates with the proneural bHLH transcription factor, Neurog2, to orchestrate regional gene expression profiles in the developing cortex [39]. In addition, Bmp5 is a gradient morphogen also involved in neuronal specification in the dorsal forebrain [86]. In contrast, Dlx4 is a homeobox protein that exists in a bigene cluster with Dlx3, which is expressed in the ventral forebrain [87]. These cumulative results suggest that REST and CoREST orchestrate neuronal subtype specification through effects on transcription factors with important roles in both dorsal and ventral regional patterning.

### Association of Promoter Occupancy with Developmental Gene Expression Profiles

To assess the potential functional roles played by REST and CoREST in neuronal developmental gene expression programs, we also correlated profiles for REST and CoREST promoter occupancy with differential gene expression patterns. Specifically, we evaluated the developmental transitions of the four neuronal subtypes from their most proximate progenitors: different NSC species into mature neuronal subtypes (CHOLNs and MSNs), radial glia (RG) into GLUTNs, and bipotent neuronal-OL progenitors (N/OPs) into GABANs (Table 3).

**Table 3:** Comparative analysis of REST and CoREST promoter occupancy and corresponding gene expression profiles during neuronal developmental transitions.

		REST	CoREST
*A*	*B*	*+/−*	*+/−*
**NSC**	**MSN**		
No	Yes	28/53	38/51
Yes	No	75/30	114/139
**NSC**	**CHOLN**		
No	Yes	69/40	64/19
Yes	No	49/22	138/67
**RG**	**GLUTN**		
No	Yes	58/20	39/4
Yes	No	25/27	41/21
**N/OP**	**GABAN**		
No	Yes	118/160	233/218
Yes	No	15/29	42/44

We evaluated specification of neuronal subtypes from their most proximate progenitors, including the developmental transition of specific NSC species into their corresponding mature neuronal subtypes (CHOLNs and MSNs), of radial glia (RG) into GLUTNs, and of bipotent neuronal-OL progenitors (N/OPs) into GABANs. The absence or presence of REST and CoREST promoter occupancy for target genes within each cell type are indicated by no and yes, respectively. Each pair of numbers represents genes up regulated and down regulated (+/−), respectively, during the transition from the proximal progenitor (column A) to its immediate progeny (column B).

We identified genes that displayed either gain or loss of REST or CoREST promoter occupancy and also exhibited differential expression between the two cell types comprising the developmental transition. Among these, we found subsets of genes with gain of REST or CoREST promoter occupancy during the transitions that were associated with both gene activation and repression. Conversely, we also identified subsets of genes with loss of REST or CoREST promoter occupancy during the transitions that were similarly associated with both gene activation and repression. For example, among genes where REST was bound in NSCs but not bound in MSNs, 75 genes were up regulated and 30 were down regulated in MSNs. Also, among genes where CoREST was bound in NSCs but not bound in CHOLNs, 138 genes were up regulated and 67 were down regulated in CHOLNs. In contrast, among genes where REST was not bound in N/OPs but bound in GABANs, 118 genes were up regulated and 160 were down regulated in GABANs. Among genes where CoREST was not bound in NSCs but bound in MSNs, 114 genes were up regulated and 139 were down regulated in MSNs. Our cumulative results suggest that REST and CoREST modulate NSC-mediated neuronal subtype specification through different profiles of REST and CoREST promoter occupancy that mediate a complex repertoire of gene expression programs involved in neuronal homeostasis, cell cycle progression, apoptosis, and neuronal subtype specific transcriptional processes.

## Discussion

The developmental fates of stem and progenitor cells within the nervous system are determined by a complex series of spatial and temporal extracellular cues which induce selective classes of cross-repressive patterning genes and combinatorial transcription factor codes that ultimately lead to cellular functional diversification. We interrogated these molecular mechanisms and found that REST and CoREST appear to play key roles in orchestrating neuronal subtype specific gene expression programs. By characterizing corresponding profiles of genome-wide REST and CoREST promoter occupancy and gene expression in a number of terminally differentiated neuronal subtypes (e.g., CHOLNs, GABANs, GLUTNs, and MSNs), we identified neuronal subtype specific REST and CoREST “regulons” [4] which seem to be important for promoting features associated with a particular neuronal subtype while concurrently repressing characteristics of alternative neural cell types. These observations support the conclusion that the highly selective REST and CoREST regulons that we uncovered mediate neuronal subtype specification.

Consistent with previous reports, the REST and CoREST target genes that we uncovered encode a broad array of factors, including those which define cell identity and connectivity (e.g., transcription factors, gradient morphogens and cytokines, cell surface molecules, neurotransmitter receptors, and synaptic vesicle proteins) and modulate homeostasis (e.g., metabolic factors, DNA repair enzymes, and cell cycle regulators) [88], [89], [90], [91]. REST and CoREST targeted genes that encode members of key neural developmental signaling pathways, such as FGF, RA, EGF, Notch, BMP, SHH, and WNT. They also bound to the promoters of genes with important neuronal subtype specific roles in CHOLNs (e.g., *Bmp2*, *Smad2*, *Lhx8*, and *Cnr1*), GABANs (e.g., *Bhlhb5*, *Rarβ*, and *Elavl2*), GLUTNs (e.g., *Tlx1*, *Bmp2*, and *Lif*), and MSNs (e.g., *Nts*, *Grm5*, and *Mash1*). Together, these findings are consistent with emerging evidence demonstrating that REST and CoREST complexes can act with high degrees of context-specificity depending on developmental stage, cell type, and gene locus. While functions of REST have previously been examined in ESCs, certain populations of NSCs, and other cell types, this is the first study to link differential deployment of REST and CoREST to neuronal subtype specification, and it significantly expands our understanding of the mechanisms that mediate this critical process.

Recent reports have suggested that the properties of neuronal subtypes are sculpted by trans-acting terminal selector genes (TSGs), which modulate the expression of cis-regulatory terminal selector motif (TSM) associated genes [90]. In this model, TSGs, such as transcription factors, specify neuronal subtype identity by modulating terminal differentiation gene expression through interactions with their cognate TSMs contained within neuronal subtype specific genes [90]. Despite the recognition of this terminal regulatory logic, the molecular circuitry that explains how different neuronal subtype specific gene networks are dynamically constrained by other types of selector genes has yet to be elucidated.

Like TSGs, REST and CoREST are important nodes in the regulatory circuitry underlying the generation of neuronal diversity, though they have broader effects on neuronal identity and function by controlling a larger cohort of positively and negatively interacting epigenetic modulators, transcription factors, and neuronal differentiation genes. While REST and CoREST are distinct from TSGs, they seem to share a number of properties, and therefore, we suggest that they act as “facultative selector genes” (FSGs). This role for REST and CoREST in orchestrating neuronal subtype specification is supported by their regulation of RE1-associated neuronal target genes as well as additional molecular mechanisms. First, TSGs can function either alone or in synergistic combination with each other [90], and previous work as well as our current findings indicate that REST and CoREST can function both independently or in tandem as members of a combinatorial complex [10], [12], [13]. Second, TSGs perform maintenance functions throughout the life of a neuron in order to maintain the post-mitotic, differentiated state [90]. Our results indicate that both REST and CoREST are expressed in the nucleus of all neuronal subtypes examined, suggesting roles in the maintenance of terminally differentiated neuronal subtypes. Also, the function of TSGs is orchestrated by positive autoregulation (i.e. TSGs maintain their own expression), a process that may mediate REST function because of the presence of an RE1 in the promoter of the REST gene [92], [93]. TSGs can also repress alternative cell fates by repressing selector genes for other cell types [90]. Our data support a similar role for REST and CoREST as these factors modulate glial genes in neuronal subtypes (e.g., *Mobp*). Moreover, a number of REST and CoREST targets encode factors that repress alternative neuronal fates (e.g., *Lhx8* in CHOLN, *Bhlhb5* in GABAN, and *Tlx1* in GLUTN), suggesting that REST and CoREST mediate the functional properties of specific neuronal subtypes in a myriad of ways. Finally, our results indicate that the majority of REST and CoREST target genes are unique for each neuronal subtype (Figures 3 and 4), suggesting that a REST and CoREST mediated generic neuronal differentiation program does not exist. Together, these observations imply that REST and CoREST function as FSGs that act upstream of TSGs and have a broad range of effects on neuronal gene networks in order to promote the acquisition and maintenance of neuronal subtype identity while repressing alternative cellular fates [90].

A more detailed understanding of the properties of REST and CoREST transcriptional networks and their dynamic functions is necessary to further characterize the regulatory logic underlying neuronal subtype specification and also to elucidate their potential roles in diseases with selective vulnerability of neuronal subtypes to neurodegeneration. For example, Huntington's disease (HD) is caused by a polyglutamine expansion repeat in Huntingtin (Htt) and characterized, in part, by abnormal REST trafficking and transcriptional dysregulation. We found that REST and CoREST specifically target genes encoding a number of Htt interacting proteins in distinct neuronal subtypes (CHOLNs, GABANs, and GLUTNs but not MSNs) including Hip2, which is a ubiquitin conjugating enzyme involved in polyglutamine induced cell death, Ift57 (Hippi), which regulates apoptosis, and Prpf40b, which is a spliceosomal factor also associated with methyl CpG binding protein 2 (Mecp2) and Rett syndrome. Neuronal subtype specific REST and CoREST targeting of these factors may be important for mediating the selective vulnerability of certain neuronal subpopulations in HD. Potential roles for REST and CoREST in modulating the specific profiles of neuronal loss in neurodegenerative diseases are further suggested by our finding that REST and CoREST targeted a number of Alzheimer's disease associated genes. These included *Blmh*, *Lrp1*, and *ApoE*, in CHOLN, and *A2m*, *Appbp1*, *Abca2*, *Sorl1*, and *Icam5* in other neuronal subtypes. Moreover, REST and CoREST selectively targeted genes that, when mutated, cause a broader array of diseases characterized by varying patterns of neurodegeneration including spinocerebellar ataxias (e.g., *Atxn7*, *Fgf14*, *Atxn2*, and *Tbp*), spastic paraplegias (e.g., *Spg7*, *Nipa1*, *Kif5a*, and *Spg20*), Parkinson's disease (e.g., *Park2* and *Snca*), frontotemporal dementia (e.g., *Mapt*), myoclonic and dopa-responsive dystonias (e.g., *Drd2* and *Gch1*), pantothenate kinase associated neurodegeneration (e.g., *Pank2*), and hemochromataosis (e.g., *Hfe*).

In this study, we suggest that REST and CoREST are responsible for integrating the intrinsic mechanisms (e.g., transcription factor codes and epigenetic modifications) and extrinsic cues (e.g., growth factors, cytokines, and extracellular signaling pathways), which maintain the fidelity, diversity, and plasticity of neuronal subtypes. Uncovering the molecular codes that determine neuronal subtype identity and function will likely add to our understanding of normal CNS processes and contribute to the characterization of neurological diseases involving deregulation of specific regional neuronal subtypes.

## Materials and Methods

### Cell Cultures

Cultures preparations were generated as previously described with minor modifications [22], [94], [95], [96], [97], [98]. Briefly, multipotent and more lineage restricted progenitor species derived from embryonic day 14.5 (E14.5) ventral forebrain regions of CD1 mice were plated and propagated in serum-free media (SFM) composed of DMEM/F12 (GIBCO) containing B27 and N2 (GIBCO) with the addition of specified factors for various time intervals and subsequently examined by immunofluorescence microscopy to define neural lineage profiles [22], [94], [95], [96], [97], [98], by Western blot analysis to detect REST and CoREST protein expression [95], [99], and by QChip and ChIP-chip to identify DNA binding sites for REST and CoREST as previously described [100], [101], [102]. Primary neural stem cell (NSC) clones were generated by application of basic fibroblast growth factor (bFGF, 10 ng/ml) for 7 days in vitro (DIV) and then dissociated using 0.05% trypsin (GIBCO) for 15 minutes at 37°C. Individual cells were re-propagated in bFGF for an additional 2 DIV to form secondary NSC clones that were used for experiments and are referred to herein as NSCs. The expansion of secondary NSC clones was limited to 2 DIV to avoid inclusion of differentiated neural species from this culture condition. This culture paradigm eliminates intermediate neural progenitor species and other proliferating neural developmental cell types present in primary NSC clones. Lineage-restricted neuronal-OL progenitor (N/OP) clones were generated from dissociated primary NSC clones by application of bFGF and the N-terminal active form of Shh (N-Shh, 100 ng/ml) for 2 days in vitro (2 DIV) [22]. GABAergic neurons (GABANs) were generated by application of BMP2 (10 ng/ml) to N/OPs for 2 DIV [22] propagated on poly-D-lysine (PDL) coated culture dishes with the addition of laminin (3 µg/ml, BD Biosciences). Cholinergic neurons (CHOLNs) were generated from E14.5 ventral forebrain-derived NSCs by minor modification of a previously published method [103]. Briefly, NSCs were dissociated in 0.05% trypsin for 15 minutes at 37°C and re-propagated in Neurobasal medium (GIBCO) supplemented with N2 on PDL coated culture dishes with the addition of laminin (3 µg/ml), bFGF (10 ng/ml), N-Shh (100 ng/ml) and nerve growth factor (NGF, 200 ng/ml) for 2 DIV followed by application of N-Shh and NGF (same dose) for an additional 14 DIV. Medium spiny projection neurons (MSNs) were also generated from E14.5 ventral forebrain derived NSCs by modifying a previously published method [104]. Dissociated NSCs were plated on PDL-coated cultures dishes containing Neurobasal, B27 and laminin (3 µg/ml) and were treated with bFGF (10 ng/ml), N-Shh (50 ng/ml) and brain derived neurotrophic factor (BDNF, 50 ng/ml) for 1 DIV followed by application of BDNF (100 ng/ml) for an additional 14 DIV. Media preparations containing NGF and BDNF were replenished every 3 DIV for CHOLNs and MSNs, respectively. Glutamatergic neurons (GLUTNs) were generated directly from radial glial cells (RG) that were obtained from dorsal forebrain derived primary NSC clones. Briefly, individual cells dissociated from dorsal forebrain derived NSC clones were propagated in SFM on PDL-coated dishes in SFM supplemented with laminin (3 µg/ml) in the presence of bFGF (10 ng/ml) and LIF (10 ng/ml) for 2 DIV to elaborate RG. GLUTN were subsequently generated by withdrawal of bFGF and Shh from RG for an additional 4 DIV.

For comparative analysis, OL precursor cells (OLpres) were obtained from N/OP clones by application of platelet-derived growth factor (PDGF-AA, 10 ng/ml) for 2 DIV. Sequential developmental stages of the OL lineage were obtained by propagation in SFM containing laminin (3 µg/ml, BD Biosciences) on poly-D-lysine (PDL) coated culture dishes. OL progenitors (OLpros) were obtained from OL precursor species by application of PDGF-AA (10 ng/ml) for an additional 2-4 DIV. Post-mitotic OLs (pmOLs) and myelinating OLs (myOLs) were obtained from OL progenitors by withdrawal of PDGF-AA for an additional 2 and 4 DIV, respectively [105]. Astrocytes (ASs) were obtained by dissociation of NSC clones using 0.05% trypsin (Sigma) for 15 minutes at 37°C and re-propagation of individual cells in epidermal growth factor (EGF) for 7 DIV with subsequent addition of bone morphogenetic protein 2 (BMP2) for 5 DIV [97], [98]. We followed institutional IACUC guidelines for experiments in which primary mouse tissue specimens were used.

**Immunofluorescence microscopic analysis** was performed as we have previously described [95], [96]

### Specific Antibody Preparations

All antibodies exhibited selective immunoreactivity for mouse cells and tissue sections, and each antibody exhibited a complete absence of alternate cross-reactivity. The following antibodies were utilized: CoREST, REST, and normal rabbit IgG (1∶100, Upstate, Temecula, CA, USA), the neuroepithelial marker (nestin, mIgG1, 1∶200, Pharmingen), N/OP markers (Olig2, goat IgG, 1∶300, R&D and Mash1, mIgG1, 1∶100, Pharmingen), neuronal marker (β Tubulin III, mIgG2b, 1∶700, Sigma), GABAN marker (GABA, rIgG, 1∶1000, Sigma), GLUTN marker (Glutamate, rIgG, 1∶1000, Sigma), CHOLN marker (ChAT, goat IgG, 1∶100, Millipore), MSN marker (DARPP32, rIgG, 1∶100, Santa Cruz). Isotype specific secondary antibodies were utilized at 1∶1500 dilution according to the required fluorophore combinations (Invitrogen). Secondary antibodies utilized for Western blot analysis were HRP conjugated (GE Healthcare).

### Western Blot Analysis

Cell cultures were processed for Western blot analysis as described previously [95], [99], [106], [107]. Briefly, cells were homogenized in nine volumes of buffer comprising 0.32 m sucrose, 50 mm Tris-HCl, pH 8.0, EDTA-free protease inhibitors cocktail (Roche) and 0.5 mm phenylmethylsulfonyl fluoride using a glass–Teflon homogenizer (10 strokes at 800 rpm) on ice and centrifuged at 900 g for 10 min and lysed in sodium dodecyl sulfate sample-loading buffer for Western blot analysis.

### Growth Factor Preparations

To generate the various neural stem, progenitor and more differentiated neuronal subtype species the following growth factor preparations were utilized: recombinant bFGF (Collaborative Biomedical Products), N-Shh and recombinant mouse β-NGF (R&D Systems), human BDNF (BioVision), BMP2 (gift from Genetics Institute) and LIF (Chemicon). To generate the various complementary and comparative glial species the following additional growth factor preparations were utilized: recombinant EGF and PDGF-AA (R&D Systems).

### Quantitative Chromatin Immunoprecipitation (QChIP)

Cultures were prepared essentially as previously described [22], [94] and used in chromatin immunoprecipitations (ChIP) [100], [101], [102], with slight modifications. Between 1×10^6^ and 3×10^6^ cells were used per antibody. The following antibodies were utilized: CoREST, REST, and normal rabbit IgG (Upstate, Temecula, CA, USA). An additional control included the absence of antibody (input chromatin). Antibodies were first validated using a peptide competition assay. For 1×10^6^ cells, 10 µg of antibody was used. Enrichment of fragments by ChIP was quantified by using 1 µl of ChIP product for real-time PCR using the SYBR® Green kit (Applied Biosystems, Foster City, CA, USA) in a 7000 Real Time PCR system® (Applied Biosystems, CA, USA). Validated CoREST and REST promoter binding sites were used as positive controls, while a non-target gene promoter was used as a negative control. ΔC_T_ values were obtained using the ΔΔC_T_ method [108]. For validation of ChIP-chip results, primers, 50 to 150 base pairs in length, were designed to flank the binding peak within each of the respective promoter sequences.

### ChIP-Chip Assays

To investigate the specific roles of CoREST and REST throughout these early neural developmental stages, we examined the differential binding profiles of CoREST and REST by determining its gene-specific targets through a series of ChIP-chip experiments. Chromatin immunoprecipitation (ChIP) is an *in vivo* technique that can be used to identify transcription factor binding sites. To determine that the CoREST and REST antibodies were specific for known targets, ChIP was first performed in an Oli-Neu cell line [109] with a CoREST antibody, REST antibody, control IgG, or no antibody (input). Samples were then analyzed by quantitative PCR (QPCR) using primers specific for both a known and previously validated CoREST and REST target gene, GluR2, and a negative control [12], [110]. Chromatin immunoprecipitation targets were determine by utilizing a fold-enrichment greater than 1.5. We then performed a series of ChIP-chip experiments in order to build a comprehensive profile of CoREST and REST targets throughout early neural developmental stages. The mouse promoter array was based on MM8/mouse genome build 36 from February 2006. The design is based only on normal RefSeq genes (17,355 genes) and includes 2,000 base pairs upstream of the transcriptional start sites and 500 base pairs downstream. The probe size ranges from 50–75 base pairs and the spacing interval is 100 base pairs.

### ChIP-Chip Data Analysis

Analysis was performed essentially as previously described [111]. Enrichment was calculated for each probe by computing the log-ratio value for the ChIP immunoprecipitated product in comparison to the input chromatin. For all ChIP-chip experiments, in order to find promoter peaks, a maximum log-ratio value for a window consisting of three consecutive probes was determined for both experimental data and a random permutation of the data. A positive threshold was then established to determine the probability for real enrichment. This positive threshold was determined by examining signals of known CoREST and REST binding sites, GluR2, and calbindin, respectively [12], [110]. A 90% positive threshold was used. The gene target lists were generated based on the intersection of genes across a minimum of two arrays for each experimental paradigm. We validated these ChIP-chip results in a representative sample of cell types with QChIP and found 83% and 94% correlation for CoREST and REST, respectively. These results indicate that the ChIP-chip technique and data analysis methods we used to characterize CoREST and REST target genes are effective approaches for identifying valid binding sites.

### Functional Classification of Target Genes

Target genes were functionally annotated through the application of Ingenuity Pathways Analysis (Ingenuity® Systems, www.ingenuity.com).

### Gene Expression Assays and Analysis

The gene expression array was based on MM8/mouse genome build 36 from February 2006 (NimbleGen, Madison, WI). The Robust Multi-Array Average (RMA) algorithm was used and data was also analyzed by the significant analysis of microarray methods (SAM). Each of the 11 different cell types was compared to neural stem cells. Three biological replicates were conducted for each cell type.

## Supporting Information

Figure S1 Identification of neural stem cells (NSCs) and their more lineage restricted progeny that give rise to selective dorsal and ventral neuronal subtypes characterized by the expression of selective neural lineage markers and developmental stage specific transcription factors. Immunofluorescence microscopy of expression profiles for neural lineage markers and transcription factors in NSCs derived from dorsal and ventral mouse forebrain, and progressively maturing intermediate neural progenitors and neuronal subtype species. The neuroepithelial marker, nestin (FITC) is expressed by NSCs, radial glia (RG), and neuronal-oligodendrocyte progenitors (N/OPs). RC2 (Cy5/TRITC) expression is limited to radial glial cells. The basic helix-loop-helix transcription factors, Mash1 (FITC) and Olig2 (TRITC) are only expressed by N/OPs (Nestin-Mash1-Olig2 co-expression studies cannot be performed due to antibody isotype incompatibility). Dorsal (glutamatergic-, GLUTN) and ventral (GABAergic-, GABAN; medium spiny projection-; MSN and cholinergic-, CHOLN) neurons were identified by complete overlap of the early neuronal lineage marker, beta-tubulin III with subtype specific neurotransmitters, or substrate/enzymes involved in the synthesis of those neurotransmitters (glutamate, GABA, DARPP32 and ChAT).
(3.53 MB TIF)

Figure S2 Expression and subcellular localization of REST in neural stem cells (NSCs) and more lineage restricted progeny that give rise to selective dorsal and ventral forebrain derived neuronal subtypes. Immunofluorescence microscopy of REST (TRITC) expression profiles in NSCs, lineage restricted intermediate neural progenitor species and their progeny composed of a selective subset of ventral and dorsal forebrain neuronal species. REST is expressed in the nucleus of all undifferentiated NSCs and intermediate neural progenitors including radial glia (RG) and neuronal-oligodendrocyte progenitors (N/OP). REST expression is noted in both nuclear and cytoplasmic compartments in mature dorsal (glutamatergic-; GLUTN) and ventral (GABAergic-, GABAN; medium spiny projection-, MSN; and cholinergic-, CHOLN) neuronal species. Antibodies to specific markers for NSCs, RGs, and CHOLN (FITC) were used to identify distinct stages of cellular maturation. Due to the absence of a specific lineage marker, N/OPs were labeled with the NSC marker, nestin (FITC), but selectively identified by the presence of two bHLH transcription factors, Olig2 and Mash1 (Figure S1), while GLUTN, GABAN and MSN were labeled with the early neuronal marker, beta-tubulin III (FITC) because of the isotype incompatibility between the REST antibody and neuronal subtype specific markers. The complete overlap of beta-tubulin III with these neuronal subtype specific markers is documented in Figure S1. Scale bars = 100 µm.
(3.76 MB TIF)

Figure S3 Expression and subcellular localization of CoREST in neural stem cells (NSCs) and more lineage restricted progeny that give rise to selective dorsal and ventral forebrain derived neuronal subtypes. Immunofluorescence microscopy of CoREST (TRITC) expression profiles in NSCs, lineage restricted intermediate neural progenitor species and more mature ventral and dorsal forebrain neuronal species. CoREST is expressed in the nucleus of all undifferentiated NSCs and intermediate neural progenitors including radial glia (RG) and neuronal-oligodendrocyte progenitors (N/OP) as well as dorsal (glutamatergic; GLUTN) and ventral (GABAergic-, GABAN; medium spiny projection-, MSN; and cholinergic-, CHOLN) forebrain derived neuronal species. Antibodies specific to NSCs, RG, and CHOLN (FITC) were used to identify selective cellular developmental stages. N/OPs were labeled with nestin (FITC) as well as Olig2/Mash1 (Figure S1), GLUTN, GABAN and MSN were co-labeled with the early neuronal marker, beta-tubulin III (FITC) to avoid isotype incompatibility of CoREST antibody with neuronal subtype specific markers (Figure S1). Scale bars = 100 µm.
(4.23 MB TIF)

Figure S4 Western blot analysis of REST and CoREST expression in NSCs, intermediate progenitors and mature neuronal subtypes. REST and CoREST are ubiquitously expressed in all cell types examined in our developmental paradigm.
(0.46 MB TIF)

Table S1 Composite profiles of REST and CoREST target genes in individual neuronal subtypes.
(1.69 MB XLS)

Table S2 Selective profiles of REST and CoREST target genes encoding epigenetic factors in individual neuronal subtypes.
(0.09 MB DOC)

Table S3 Selective profiles of REST and CoREST target genes encoding cell cycle factors in individual neuronal subtypes.
(0.08 MB DOC)

Table S4 Selective profiles of REST and CoREST target genes encoding ubiquitin-proteasome factors in individual neuronal subtypes.
(0.07 MB DOC)

Table S5 Selective profiles of REST and CoREST target genes encoding apoptosis and cell viability factors in individual neuronal subtypes.
(0.05 MB DOC)

Table S6 Selective profiles of REST and CoREST target genes encoding neuronal identity factors in individual neuronal subtypes.
(0.10 MB DOC)
