# Supplementary figures and images for: REST and CoREST Modulate Neuronal Subtype Specification, Maturation and Maintenance
Source: PLoS One. 2009 Dec 7;4(12):e7936. doi: 10.1371/journal.pone.0007936 (PMC2782136; doi:10.1371/journal.pone.0007936)

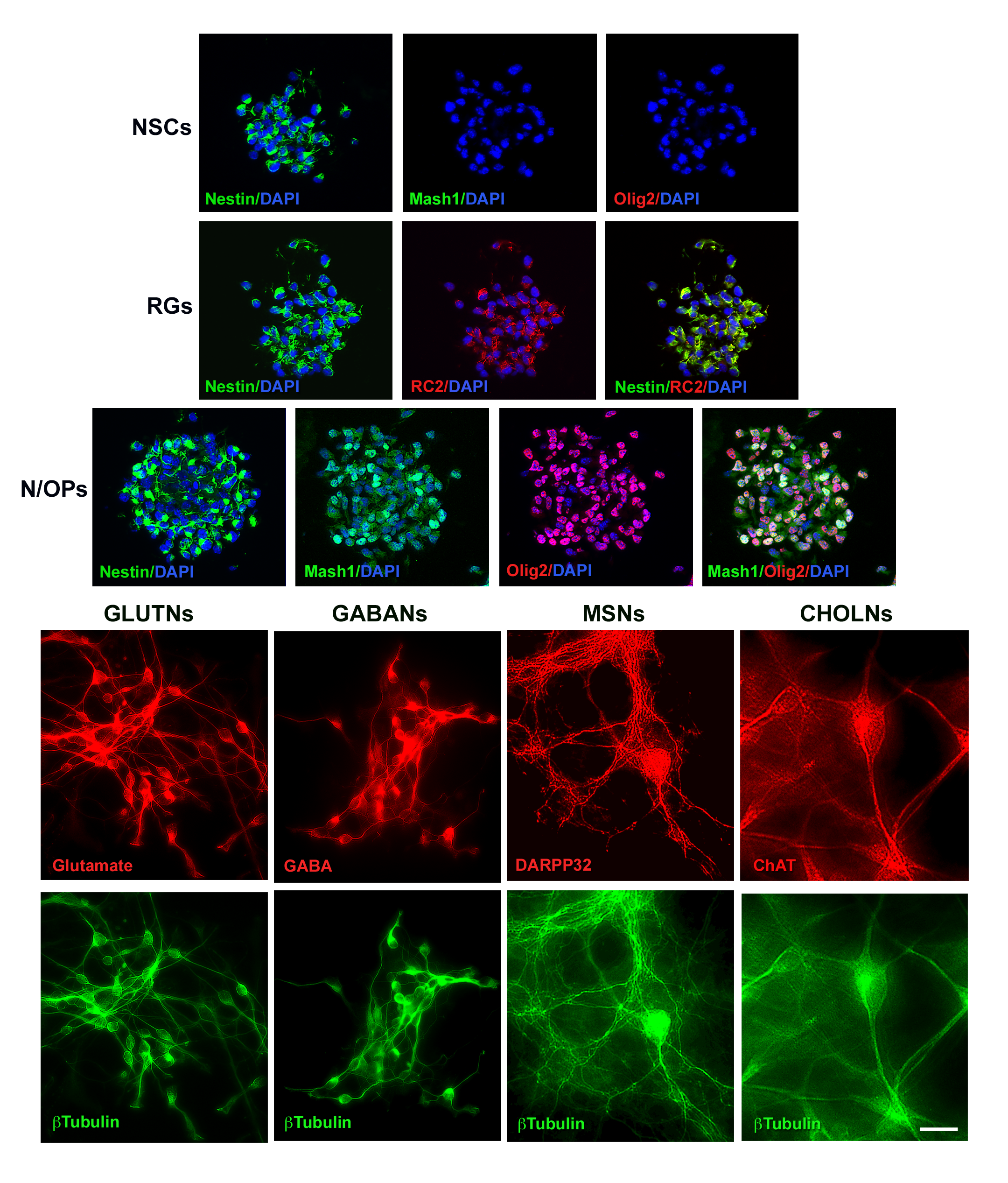

Supplement: Figure S1 — Identification of neural stem cells (NSCs) and their more lineage restricted progeny that give rise to selective dorsal and ventral neuronal subtypes characterized by the expression of selective neural lineage markers and developmental stage specific transcription factors. Immunofluorescence microscopy of expression profiles for neural lineage markers and transcription factors in NSCs derived from dorsal and ventral mouse forebrain, and progressively maturing intermediate neural progenitors and neuronal subtype species. The neuroepithelial marker, nestin (FITC) is expressed by NSCs, radial glia (RG), and neuronal-oligodendrocyte progenitors (N/OPs). RC2 (Cy5/TRITC) expression is limited to radial glial cells. The basic helix-loop-helix transcription factors, Mash1 (FITC) and Olig2 (TRITC) are only expressed by N/OPs (Nestin-Mash1-Olig2 co-expression studies cannot be performed due to antibody isotype incompatibility). Dorsal (glutamatergic-, GLUTN) and ventral (GABAergic-, GABAN; medium spiny projection-; MSN and cholinergic-, CHOLN) neurons were identified by complete overlap of the early neuronal lineage marker, beta-tubulin III with subtype specific neurotransmitters, or substrate/enzymes involved in the synthesis of those neurotransmitters (glutamate, GABA, DARPP32 and ChAT). (3.53 MB TIF) [file pone.0007936.s001.tif]

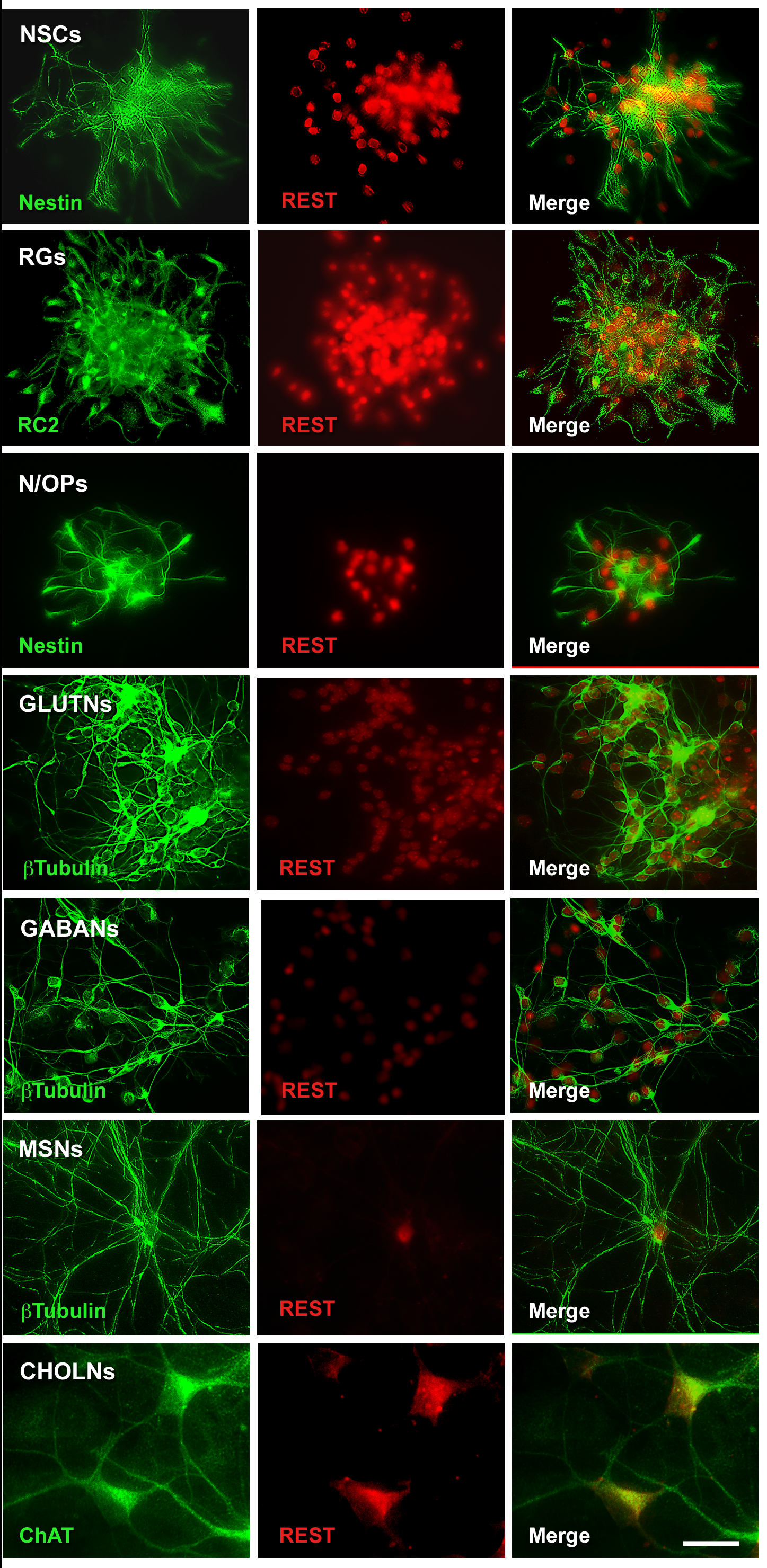

Supplement: Figure S2 — Expression and subcellular localization of REST in neural stem cells (NSCs) and more lineage restricted progeny that give rise to selective dorsal and ventral forebrain derived neuronal subtypes. Immunofluorescence microscopy of REST (TRITC) expression profiles in NSCs, lineage restricted intermediate neural progenitor species and their progeny composed of a selective subset of ventral and dorsal forebrain neuronal species. REST is expressed in the nucleus of all undifferentiated NSCs and intermediate neural progenitors including radial glia (RG) and neuronal-oligodendrocyte progenitors (N/OP). REST expression is noted in both nuclear and cytoplasmic compartments in mature dorsal (glutamatergic-; GLUTN) and ventral (GABAergic-, GABAN; medium spiny projection-, MSN; and cholinergic-, CHOLN) neuronal species. Antibodies to specific markers for NSCs, RGs, and CHOLN (FITC) were used to identify distinct stages of cellular maturation. Due to the absence of a specific lineage marker, N/OPs were labeled with the NSC marker, nestin (FITC), but selectively identified by the presence of two bHLH transcription factors, Olig2 and Mash1 (Figure S1), while GLUTN, GABAN and MSN were labeled with the early neuronal marker, beta-tubulin III (FITC) because of the isotype incompatibility between the REST antibody and neuronal subtype specific markers. The complete overlap of beta-tubulin III with these neuronal subtype specific markers is documented in Figure S1. Scale bars = 100 µm. (3.76 MB TIF) [file pone.0007936.s002.tif]

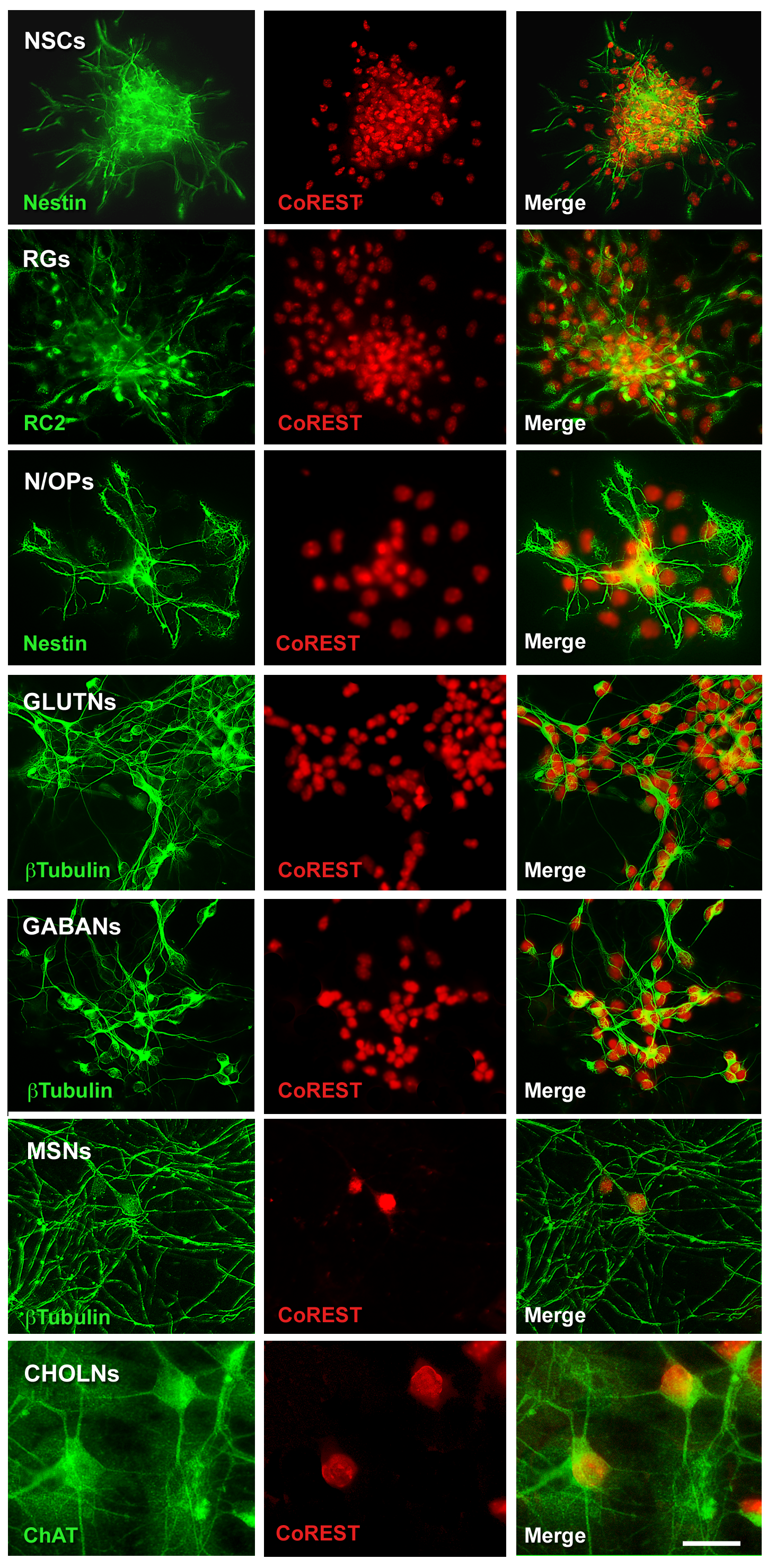

Supplement: Figure S3 — Expression and subcellular localization of CoREST in neural stem cells (NSCs) and more lineage restricted progeny that give rise to selective dorsal and ventral forebrain derived neuronal subtypes. Immunofluorescence microscopy of CoREST (TRITC) expression profiles in NSCs, lineage restricted intermediate neural progenitor species and more mature ventral and dorsal forebrain neuronal species. CoREST is expressed in the nucleus of all undifferentiated NSCs and intermediate neural progenitors including radial glia (RG) and neuronal-oligodendrocyte progenitors (N/OP) as well as dorsal (glutamatergic; GLUTN) and ventral (GABAergic-, GABAN; medium spiny projection-, MSN; and cholinergic-, CHOLN) forebrain derived neuronal species. Antibodies specific to NSCs, RG, and CHOLN (FITC) were used to identify selective cellular developmental stages. N/OPs were labeled with nestin (FITC) as well as Olig2/Mash1 (Figure S1), GLUTN, GABAN and MSN were co-labeled with the early neuronal marker, beta-tubulin III (FITC) to avoid isotype incompatibility of CoREST antibody with neuronal subtype specific markers (Figure S1). Scale bars = 100 µm. (4.23 MB TIF) [file pone.0007936.s003.tif]

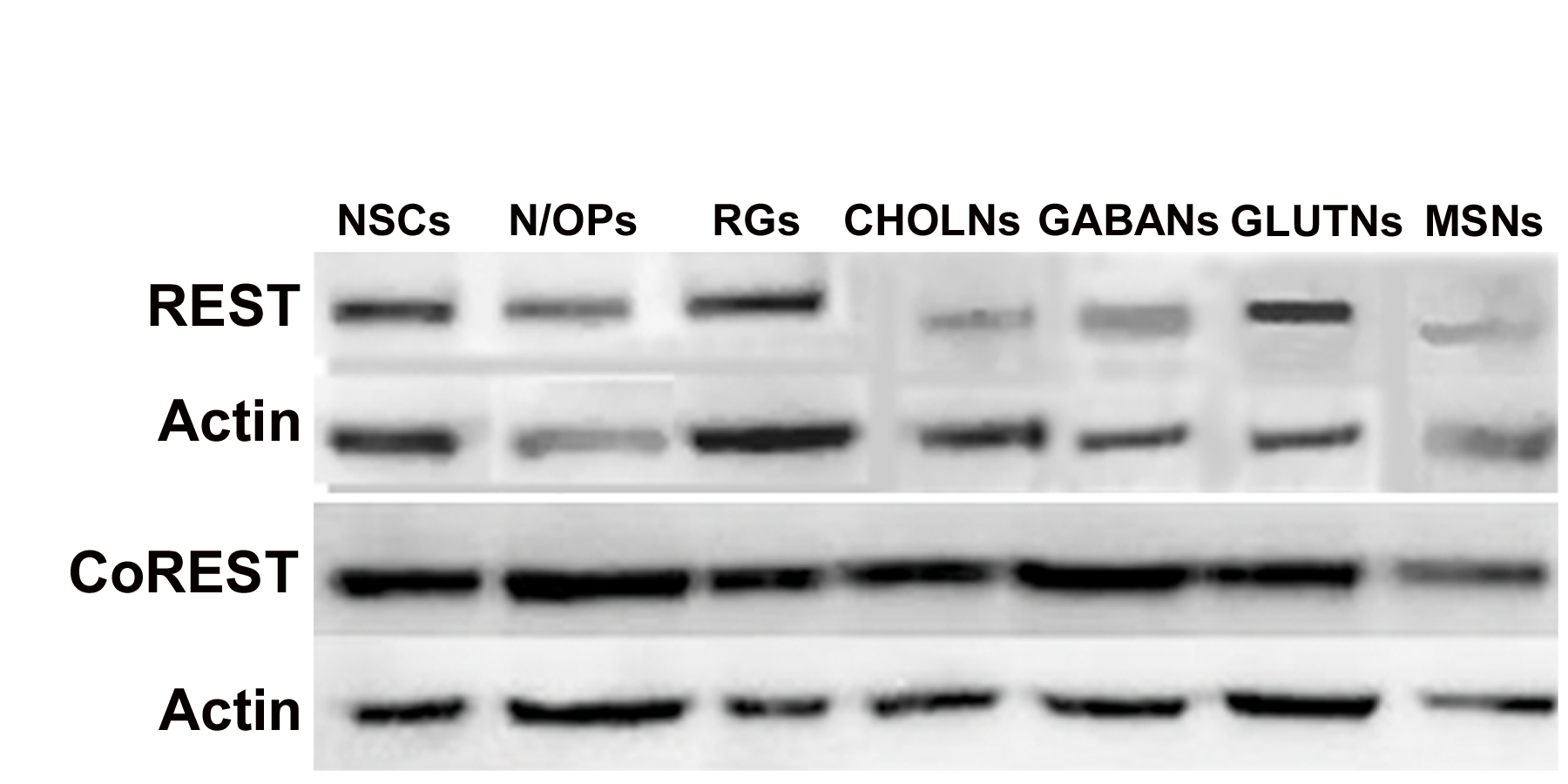

Supplement: Figure S4 — Western blot analysis of REST and CoREST expression in NSCs, intermediate progenitors and mature neuronal subtypes. REST and CoREST are ubiquitously expressed in all cell types examined in our developmental paradigm. (0.46 MB TIF) [file pone.0007936.s004.tif]
