# Supplementary material for: REST and CoREST Modulate Neuronal Subtype Specification, Maturation and Maintenance
Source: PLoS One. 2009 Dec 7;4(12):e7936. doi: 10.1371/journal.pone.0007936 (PMC2782136; doi:10.1371/journal.pone.0007936)
Supplement: Table S2 — Selective profiles of REST and CoREST target genes encoding epigenetic factors in individual neuronal subtypes. (0.09 MB DOC) [file pone.0007936.s006.doc]

|  | **REST** | | | | **CoREST** | | | |
| --- | --- | --- | --- | --- | --- | --- | --- | --- |
| **Gene** | **CHOLNs** | **GABANs** | **GLUTNs** | **MSNs** | **CHOLNs** | **GABANs** | **GLUTNs** | **MSNs** |
| Hist1h4k | 0 | 0 | 1 | 0 | 1 | 0 | 0 | 1 |
| Mcm8 | 1 | 0 | 0 | 0 | 0 | 1 | 0 | 1 |
| Hist1h2bp | 0 | 0 | 0 | 0 | 0 | 0 | 1 | 1 |
| Hp1bp3 | 0 | 0 | 0 | 0 | 0 | 1 | 0 | 1 |
| Hist1h3i | 0 | 0 | 0 | 0 | 1 | 1 | 0 | 0 |
| Jarid1d | 0 | 0 | 0 | 0 | 1 | 1 | 0 | 0 |
| Nup160 | 0 | 0 | 0 | 0 | 1 | 0 | 1 | 0 |
| Ehmt1 | 1 | 1 | 1 | 1 | 1 | 0 | 0 | 0 |
| Hist1h2be | 1 | 0 | 1 | 0 | 0 | 1 | 0 | 0 |
| Nup43 | 0 | 0 | 1 | 1 | 0 | 1 | 0 | 0 |
| Smarcc2 | 1 | 0 | 0 | 0 | 0 | 1 | 0 | 0 |
| Hist2h3c1 | 0 | 0 | 0 | 0 | 0 | 0 | 0 | 1 |
| Jarid1c | 0 | 0 | 0 | 0 | 0 | 1 | 0 | 0 |
| Cbx5 | 0 | 0 | 0 | 0 | 0 | 1 | 0 | 0 |
| Chrac1 | 0 | 0 | 0 | 0 | 1 | 0 | 0 | 0 |
| Nup210 | 0 | 0 | 0 | 0 | 0 | 0 | 0 | 1 |
| Smarca5 | 0 | 0 | 0 | 0 | 1 | 0 | 0 | 0 |
| Smarce1 | 0 | 0 | 0 | 0 | 1 | 0 | 0 | 0 |
| H3f3b | 0 | 0 | 0 | 0 | 0 | 0 | 0 | 1 |
| Hist1h1b | 0 | 0 | 0 | 0 | 0 | 0 | 0 | 1 |
| AF064553 | 0 | 0 | 0 | 0 | 0 | 1 | 0 | 0 |
| Mbd3l2 | 0 | 0 | 0 | 0 | 0 | 0 | 0 | 1 |
| Nap1l2 | 1 | 1 | 0 | 1 | 0 | 0 | 0 | 0 |
| Hist1h1c | 0 | 1 | 0 | 1 | 0 | 0 | 0 | 0 |
| Sin3b | 0 | 1 | 0 | 1 | 0 | 0 | 0 | 0 |
| Chmp4b | 1 | 0 | 0 | 1 | 0 | 0 | 0 | 0 |
| Nupl2 | 0 | 1 | 1 | 0 | 0 | 0 | 0 | 0 |
| Smc4l1 | 0 | 0 | 0 | 1 | 0 | 0 | 0 | 0 |
| Cse1l | 1 | 0 | 0 | 0 | 0 | 0 | 0 | 0 |
| Nup62 | 0 | 1 | 0 | 0 | 0 | 0 | 0 | 0 |
| Chmp5 | 1 | 0 | 0 | 0 | 0 | 0 | 0 | 0 |
| Mbd6 | 0 | 1 | 0 | 0 | 0 | 0 | 0 | 0 |
| Jmjd1a | 1 | 0 | 0 | 0 | 0 | 0 | 0 | 0 |
| Hist1h2ab | 1 | 0 | 0 | 0 | 0 | 0 | 0 | 0 |
| Nap1l3 | 0 | 0 | 0 | 1 | 0 | 0 | 0 | 0 |
| Cbx5 | 0 | 1 | 0 | 0 | 0 | 0 | 0 | 0 |
| Hist1h2bc | 1 | 0 | 0 | 0 | 0 | 0 | 0 | 0 |
| Hist1h2ac | 1 | 0 | 0 | 0 | 0 | 0 | 0 | 0 |
| Smarcc1 | 1 | 0 | 0 | 0 | 0 | 0 | 0 | 0 |
| Mizf | 0 | 1 | 0 | 0 | 0 | 0 | 0 | 0 |
| Pcgf6 | 0 | 1 | 0 | 0 | 0 | 0 | 0 | 0 |
| Mcm6 | 1 | 0 | 0 | 0 | 0 | 0 | 0 | 0 |
| H2afx | 1 | 0 | 0 | 0 | 0 | 0 | 0 | 0 |
| Hat1 | 1 | 0 | 0 | 0 | 0 | 0 | 0 | 0 |
| Aof2 | 0 | 0 | 0 | 1 | 0 | 0 | 0 | 0 |
| A630082K20Rik | 1 | 0 | 0 | 0 | 0 | 0 | 0 | 0 |
