# Supplementary material for: REST and CoREST Modulate Neuronal Subtype Specification, Maturation and Maintenance
Source: PLoS One. 2009 Dec 7;4(12):e7936. doi: 10.1371/journal.pone.0007936 (PMC2782136; doi:10.1371/journal.pone.0007936)
Supplement: Table S3 — Selective profiles of REST and CoREST target genes encoding cell cycle factors in individual neuronal subtypes. (0.08 MB DOC) [file pone.0007936.s007.doc]

|  | **REST** | | | | **CoREST** | | | |
| --- | --- | --- | --- | --- | --- | --- | --- | --- |
| **Gene** | **CHOLNs** | **GABANs** | **GLUTNs** | **MSNs** | **CHOLNs** | **GABANs** | **GLUTNs** | **MSNs** |
| Cdkal1 | 0 | 0 | 0 | 0 | 1 | 1 | 0 | 1 |
| Cdc2l1 | 1 | 0 | 0 | 1 | 1 | 0 | 0 | 1 |
| Mad2l1 | 0 | 0 | 0 | 1 | 1 | 1 | 0 | 0 |
| Cdkl4 | 0 | 0 | 0 | 0 | 0 | 1 | 0 | 1 |
| Anapc10 | 0 | 0 | 0 | 1 | 1 | 0 | 0 | 0 |
| Bcor | 0 | 1 | 0 | 0 | 1 | 0 | 0 | 0 |
| Smad2 | 0 | 0 | 1 | 0 | 1 | 0 | 0 | 0 |
| Arid4b | 1 | 0 | 0 | 0 | 0 | 0 | 0 | 1 |
| Ccna2 | 0 | 0 | 1 | 0 | 0 | 0 | 0 | 1 |
| Nucks1 | 0 | 1 | 0 | 0 | 0 | 0 | 1 | 0 |
| Mtbp | 0 | 0 | 0 | 0 | 0 | 0 | 0 | 1 |
| Mina | 0 | 0 | 0 | 0 | 0 | 1 | 0 | 0 |
| Rap2b | 0 | 0 | 0 | 0 | 0 | 0 | 0 | 1 |
| 8-Sep | 0 | 0 | 0 | 0 | 0 | 0 | 0 | 1 |
| 7-Sep | 0 | 0 | 0 | 0 | 0 | 1 | 0 | 0 |
| Tusc1 | 0 | 0 | 0 | 0 | 0 | 0 | 0 | 1 |
| Cables1 | 0 | 0 | 0 | 0 | 1 | 0 | 0 | 0 |
| Cdc14b | 0 | 0 | 0 | 0 | 0 | 0 | 0 | 1 |
| Cdc91l1 | 0 | 0 | 0 | 0 | 0 | 1 | 0 | 0 |
| 5730405I09Rik | 0 | 0 | 0 | 0 | 0 | 1 | 0 | 0 |
| Ccnj | 0 | 0 | 0 | 0 | 0 | 1 | 0 | 0 |
| Cnnm1 | 0 | 0 | 0 | 0 | 0 | 0 | 0 | 1 |
| Pak7 | 0 | 0 | 0 | 0 | 1 | 0 | 0 | 0 |
| Trp53inp2 | 0 | 1 | 0 | 1 | 0 | 0 | 0 | 0 |
| Ccni | 1 | 1 | 0 | 0 | 0 | 0 | 0 | 0 |
| Cdk5r2 | 0 | 0 | 1 | 1 | 0 | 0 | 0 | 0 |
| Cdk8 | 1 | 0 | 0 | 1 | 0 | 0 | 0 | 0 |
| Nek7 | 1 | 0 | 0 | 0 | 0 | 0 | 0 | 0 |
| Nek8 | 0 | 0 | 0 | 1 | 0 | 0 | 0 | 0 |
| Pak2 | 0 | 1 | 0 | 0 | 0 | 0 | 0 | 0 |
| Rbbp7 | 1 | 0 | 0 | 0 | 0 | 0 | 0 | 0 |
| Cdk2ap1 | 0 | 1 | 0 | 0 | 0 | 0 | 0 | 0 |
| Ccnb2 | 0 | 0 | 0 | 1 | 0 | 0 | 0 | 0 |
| Ccnb3 | 0 | 0 | 0 | 1 | 0 | 0 | 0 | 0 |
| Ccnf | 1 | 0 | 0 | 0 | 0 | 0 | 0 | 0 |
| Ccnl1 | 0 | 0 | 0 | 1 | 0 | 0 | 0 | 0 |
| Cdkn1b | 1 | 0 | 0 | 0 | 0 | 0 | 0 | 0 |
