# Supplementary material for: REST and CoREST Modulate Neuronal Subtype Specification, Maturation and Maintenance
Source: PLoS One. 2009 Dec 7;4(12):e7936. doi: 10.1371/journal.pone.0007936 (PMC2782136; doi:10.1371/journal.pone.0007936)
Supplement: Table S4 — Selective profiles of REST and CoREST target genes encoding ubiquitin-proteasome factors in individual neuronal subtypes. (0.07 MB DOC) [file pone.0007936.s008.doc]

|  | **REST** | | | | **CoREST** | | | |
| --- | --- | --- | --- | --- | --- | --- | --- | --- |
| **Gene** | **CHOLNs** | **GABANs** | **GLUTNs** | **MSNs** | **CHOLNs** | **GABANs** | **GLUTNs** | **MSNs** |
| Ubb | 0 | 1 | 0 | 0 | 0 | 1 | 0 | 1 |
| Usp9x | 0 | 0 | 1 | 0 | 1 | 0 | 0 | 1 |
| Ube2j1 | 1 | 0 | 0 | 1 | 1 | 0 | 0 | 0 |
| Ubtd1 | 0 | 0 | 0 | 1 | 0 | 1 | 0 | 0 |
| Mib1 | 0 | 1 | 0 | 0 | 0 | 1 | 0 | 0 |
| Usp26 | 0 | 0 | 1 | 0 | 1 | 0 | 0 | 0 |
| Arih1 | 0 | 0 | 0 | 0 | 0 | 1 | 0 | 0 |
| 9630054F20Rik | 0 | 0 | 0 | 0 | 1 | 0 | 0 | 0 |
| Usp2 | 0 | 0 | 0 | 0 | 0 | 0 | 0 | 1 |
| Uchl5 | 0 | 0 | 0 | 0 | 0 | 1 | 0 | 0 |
| Ube3c | 0 | 0 | 0 | 0 | 0 | 0 | 0 | 1 |
| Ube1y1 | 0 | 0 | 0 | 0 | 0 | 1 | 0 | 0 |
| Ubap2 | 0 | 0 | 0 | 0 | 0 | 0 | 0 | 1 |
| Ube4b | 0 | 0 | 0 | 0 | 1 | 0 | 0 | 0 |
| Ube2d1 | 0 | 0 | 0 | 0 | 0 | 1 | 0 | 0 |
| Usp14 | 0 | 0 | 0 | 0 | 0 | 1 | 0 | 0 |
| Usp8 | 1 | 1 | 1 | 0 | 0 | 0 | 0 | 0 |
| Ube2d2 | 0 | 1 | 1 | 0 | 0 | 0 | 0 | 0 |
| Cnot4 | 1 | 0 | 0 | 0 | 0 | 0 | 0 | 0 |
| Smurf1 | 0 | 0 | 1 | 0 | 0 | 0 | 0 | 0 |
| Usp30 | 1 | 0 | 0 | 0 | 0 | 0 | 0 | 0 |
| Usp50 | 1 | 0 | 0 | 0 | 0 | 0 | 0 | 0 |
| Usp9y | 1 | 0 | 0 | 0 | 0 | 0 | 0 | 0 |
| Ube2b | 0 | 1 | 0 | 0 | 0 | 0 | 0 | 0 |
| Ube2e1 | 1 | 0 | 0 | 0 | 0 | 0 | 0 | 0 |
| Ube2m | 1 | 0 | 0 | 0 | 0 | 0 | 0 | 0 |
| Ube2q | 0 | 1 | 0 | 0 | 0 | 0 | 0 | 0 |
| Ufm1 | 0 | 1 | 0 | 0 | 0 | 0 | 0 | 0 |
| Usp21 | 0 | 1 | 0 | 0 | 0 | 0 | 0 | 0 |
