# Supplementary material for: REST and CoREST Modulate Neuronal Subtype Specification, Maturation and Maintenance
Source: PLoS One. 2009 Dec 7;4(12):e7936. doi: 10.1371/journal.pone.0007936 (PMC2782136; doi:10.1371/journal.pone.0007936)
Supplement: Table S5 — Selective profiles of REST and CoREST target genes encoding apoptosis and cell viability factors in individual neuronal subtypes. (0.05 MB DOC) [file pone.0007936.s009.doc]

|  | **REST** | | | | **CoREST** | | | |
| --- | --- | --- | --- | --- | --- | --- | --- | --- |
| **Gene** | **CHOLNs** | **GABANs** | **GLUTNs** | **MSNs** | **CHOLNs** | **GABANs** | **GLUTNs** | **MSNs** |
| Casp1 | 0 | 0 | 0 | 0 | 0 | 0 | 1 | 1 |
| Oas1f | 0 | 0 | 0 | 0 | 0 | 0 | 0 | 1 |
| Bag4 | 0 | 0 | 0 | 0 | 0 | 0 | 0 | 1 |
| Ctsg | 0 | 0 | 0 | 0 | 0 | 0 | 0 | 1 |
| Ctsj | 0 | 0 | 0 | 0 | 0 | 0 | 0 | 1 |
| Eaf2 | 0 | 0 | 0 | 0 | 0 | 1 | 0 | 0 |
| Pdcd6ip | 0 | 0 | 0 | 0 | 0 | 1 | 0 | 0 |
| Pdcd11 | 0 | 0 | 0 | 0 | 0 | 0 | 0 | 1 |
| Oasl1 | 1 | 0 | 0 | 0 | 0 | 0 | 0 | 0 |
| Casp12 | 0 | 0 | 0 | 1 | 0 | 0 | 0 | 0 |
| Cts8 | 0 | 1 | 0 | 0 | 0 | 0 | 0 | 0 |
| Ctsf | 0 | 0 | 0 | 1 | 0 | 0 | 0 | 0 |
| Ctsr | 1 | 0 | 0 | 0 | 0 | 0 | 0 | 0 |
| Cidea | 1 | 0 | 0 | 0 | 0 | 0 | 0 | 0 |
| Pdcd2 | 0 | 1 | 0 | 0 | 0 | 0 | 0 | 0 |
| Thap3 | 0 | 1 | 0 | 0 | 0 | 0 | 0 | 0 |
