# Supplementary material for: REST and CoREST Modulate Neuronal Subtype Specification, Maturation and Maintenance
Source: PLoS One. 2009 Dec 7;4(12):e7936. doi: 10.1371/journal.pone.0007936 (PMC2782136; doi:10.1371/journal.pone.0007936)
Supplement: Table S6 — Selective profiles of REST and CoREST target genes encoding neuronal identity factors in individual neuronal subtypes. (0.10 MB DOC) [file pone.0007936.s010.doc]

|  | **REST** | | | | **CoREST** | | | |
| --- | --- | --- | --- | --- | --- | --- | --- | --- |
| **Gene** | **CHOLNs** | **GABANs** | **GLUTNs** | **MSNs** | **CHOLNs** | **GABANs** | **GLUTNs** | **MSNs** |
| Zf | 0 | 0 | 1 | 0 | 1 | 0 | 1 | 1 |
| Mbtps2 | 0 | 0 | 0 | 0 | 0 | 1 | 1 | 1 |
| Dlx4 | 0 | 0 | 1 | 0 | 0 | 1 | 0 | 1 |
| Neurod4 | 0 | 0 | 0 | 1 | 1 | 0 | 0 | 1 |
| Dmtf1 | 0 | 0 | 0 | 0 | 1 | 1 | 0 | 0 |
| Phtf1 | 0 | 0 | 0 | 0 | 1 | 1 | 0 | 0 |
| Sp3 | 0 | 0 | 0 | 0 | 1 | 0 | 1 | 0 |
| Barx1 | 0 | 0 | 0 | 1 | 0 | 1 | 0 | 0 |
| Batf | 0 | 1 | 0 | 0 | 1 | 0 | 0 | 0 |
| Bhlhb5 | 1 | 0 | 0 | 0 | 0 | 1 | 0 | 0 |
| Foxo1 | 1 | 0 | 0 | 0 | 0 | 0 | 1 | 0 |
| Hsfy2 | 0 | 1 | 0 | 0 | 0 | 1 | 0 | 0 |
| Irx4 | 1 | 0 | 0 | 0 | 0 | 0 | 0 | 1 |
| Pit1 | 0 | 0 | 1 | 0 | 0 | 1 | 0 | 0 |
| Dlx2 | 0 | 0 | 0 | 0 | 0 | 1 | 0 | 0 |
| Foxn2 | 0 | 0 | 0 | 0 | 1 | 0 | 0 | 0 |
| Gtf2a1lf | 0 | 0 | 0 | 0 | 0 | 0 | 0 | 1 |
| Gtf2h5 | 0 | 0 | 0 | 0 | 0 | 0 | 0 | 1 |
| Isl1 | 0 | 0 | 0 | 0 | 0 | 1 | 0 | 0 |
| Nfib | 0 | 0 | 0 | 0 | 1 | 0 | 0 | 0 |
| Nkx2-6 | 0 | 0 | 0 | 0 | 0 | 0 | 0 | 1 |
| Nkx6-2 | 0 | 0 | 0 | 0 | 1 | 0 | 0 | 0 |
| Pbx1 | 0 | 0 | 0 | 0 | 1 | 0 | 0 | 0 |
| Pbxip1 | 0 | 0 | 0 | 0 | 1 | 0 | 0 | 0 |
| Phtf2 | 0 | 0 | 0 | 0 | 0 | 1 | 0 | 0 |
| Pou3f4 | 0 | 0 | 0 | 0 | 0 | 0 | 1 | 0 |
| Rcor2 | 0 | 0 | 0 | 0 | 0 | 0 | 0 | 1 |
| Tcfap2d | 0 | 0 | 0 | 0 | 0 | 0 | 1 | 0 |
| Foxd1 | 0 | 0 | 1 | 1 | 0 | 0 | 0 | 0 |
| Msrb2 | 1 | 1 | 0 | 0 | 0 | 0 | 0 | 0 |
| A630018P17Rik | 0 | 1 | 0 | 0 | 0 | 0 | 0 | 0 |
| Dlx3 | 0 | 1 | 0 | 0 | 0 | 0 | 0 | 0 |
| Dmrt3 | 0 | 0 | 0 | 1 | 0 | 0 | 0 | 0 |
| Foxa3 | 1 | 0 | 0 | 0 | 0 | 0 | 0 | 0 |
| Foxc2 | 0 | 1 | 0 | 0 | 0 | 0 | 0 | 0 |
| Foxj3 | 1 | 0 | 0 | 0 | 0 | 0 | 0 | 0 |
| Gtf2a2 | 1 | 0 | 0 | 0 | 0 | 0 | 0 | 0 |
| Gtf2f1 | 1 | 0 | 0 | 0 | 0 | 0 | 0 | 0 |
| Gtf3c1 | 0 | 1 | 0 | 0 | 0 | 0 | 0 | 0 |
| Hes5 | 1 | 0 | 0 | 0 | 0 | 0 | 0 | 0 |
| Hes7 | 0 | 0 | 0 | 1 | 0 | 0 | 0 | 0 |
| Hoxb8 | 0 | 1 | 0 | 0 | 0 | 0 | 0 | 0 |
| Mlx | 1 | 0 | 0 | 0 | 0 | 0 | 0 | 0 |
| Pbx3 | 1 | 0 | 0 | 0 | 0 | 0 | 0 | 0 |
| Pou2f1 | 0 | 0 | 1 | 0 | 0 | 0 | 0 | 0 |
| Pou4f1 | 1 | 0 | 0 | 0 | 0 | 0 | 0 | 0 |
| Sox30 | 1 | 0 | 0 | 0 | 0 | 0 | 0 | 0 |
| Sox4 | 1 | 0 | 0 | 0 | 0 | 0 | 0 | 0 |
| Tcfap2c | 1 | 0 | 0 | 0 | 0 | 0 | 0 | 0 |
| Tcfap4 | 1 | 0 | 0 | 0 | 0 | 0 | 0 | 0 |
| Tfdp1 | 0 | 0 | 0 | 1 | 0 | 0 | 0 | 0 |
